# DIF-1 regulates *Dictyostelium* basal disc differentiation by inducing the nuclear accumulation of a bZIP transcription factor

**DOI:** 10.1016/j.ydbio.2011.03.024

**Published:** 2011-06-01

**Authors:** Yoko Yamada, Beatriz Nuñez-Corcuera, Jeffrey G. Williams

**Affiliations:** College of Life Sciences, Welcome Trust Building, University of Dundee, Dow St., Dundee DD1 5EH, UK

**Keywords:** Dictyostelium, bZIP, Transcription factor, DIF-1, Basal disc, *ecmB* expression

## Abstract

Exposure of monolayer *Dictyostelium* cells to the signalling polyketide DIF-1 causes DimB, a bZIPtranscription factor, to accumulate in the nucleus where it induces prestalk gene expression. Here we analyse DimB signalling during normal development. In slugs DimB is specifically nuclear enriched in the pstB cells; a cluster of vital dye-staining cells located on the ventral surface of the posterior, prespore region. PstB cells move at culmination, to form the lower cup and the outer basal disc of the fruiting body, and DimB retains a high nuclear concentration in both these tissues. In a *dimB* null (dimB−) strain there are very few pstB or lower cup cells, as detected by neutral red staining, and it is known that the outer basal disc is absent or much reduced. In the dimB− strain *ecmB*, a marker of pstB differentiation, is not DIF inducible. Furthermore, ChIP analysis shows that DimB binds to the *ecmB* promoter in DIF-induced cells. These results suggest that the differentiation of pstB cells is caused by a high perceived level of DIF-1 signalling, leading to nuclear localization of DimB and direct activation of cell type-specific gene expression.

## Research highlights

► The DimB transcription factor is nuclear enriched only in pstB cells of the slug. ► A *dimB* null lacks pstB cells and derivative tissues: the lower cup and basal disc. ► DIF induces DimB to bind to the ecmB promoter, a marker of pstB differentiation. ► DIF is the direct inducer of ecmB gene expression and pstB cell differentiation.

## Introduction

Pattern formation in multicellular organisms is normally the consequence of two intimately linked processes: cellular differentiation and morphogenetic cell movement. Cellular differentiation generally involves the sequential activation of a set of transcriptional cascades that function, successively, within a particular lineage to specify cell identity. In the major animal model systems many such cascades have been dissected in considerable detail. Morphogenetic cell movement serves to position the differentiated cells correctly within the organism but the processes involved are not as generally well understood. During *Dictyostelium* development lineage restriction is not an issue, because cell division and differentiation are in effect uncoupled, but cell movement plays a central role. It brings the initially isolated cells together and then shapes the resultant mound of cells into a slug. Later, in the process of culmination, major cell movements re-structure the slug to form a fruiting body. This comprises a tapering stalk, bearing a mass of spores, impaled into a conical basal disc that is also composed of dead, vacuolated stalk cells. The fundamental divide is the 20:80 split, between those cells that differentiate as stalk cells and those that differentiate as spore cells. However, the stalk cell precursors are of several different kinds that have different movement properties, both within the slug and at culmination (Gaudet et al., 2008).

The anterior one-fifth of the slug, the prestalk region, comprises a front half composed of pstA cells and a rear half composed of pstO cells. There are also cells scattered through the rear four-fifths of the slug, the pstO/ALC (pstO related Anterior-Like Cells), that resemble pstO cells in several ways (Gaudet et al., 2008). Most of the cells in the rear four fifths of the slug are prespore cells but there are two other populations of ALC: pstU and pstB cells. PstU cells are mainly located immediately posterior to the pstO cells and they are identified by their ability to utilise the *rta1* promoter (Yamada et al., 2010). PstB cells form a cluster on the ventral surface of the slug that is variably positioned along the length of the prespore region. PstB cells were identified by their selective staining with the vital dye neutral red (Dormann et al., 1996) and, in a parallel study, by their high level of *ecmB* expression relative to *ecmA* (Jermyn et al., 1996); the latter ratio assayed in a strain co-expressing an ecmA-gus reporter fusion and an ecmB-gal reporter fusion. Their proposed identity was based solely on a comparison of the location of the two populations. There was no direct evidence to prove that they were the same cells.

The neutral red analysis showed that the pstB cells are highly dynamic in their movement; always apposed to the ventral surface of the slug but moving back and fore through the prespore zone (Dormann et al., 1996). At culmination the vitally stained pstB cells could be seen to move to form both the lower cup and the outer basal disc. Another sub-set of prestalk cells, the pstAB cells, move downwards immediately ahead of the stalk and embed themselves into the outer basal disc, to form the inner basal disc (Sternfeld, 1992). The lower cup sits beneath the nascent spore head and seems to be necessary to help support the spore mass (Saito et al., 2008). The motive force that lifts the spores up the stalk is provided by cells located above the nascent spore mass, the upper cup cells (Sternfeld, 1998).

There is a partial understanding of the extracellular signals that induce differentiation of the various prestalk cell sub-types. DIF-1 is a polyketide produced by the prespore cells (Kay and Thompson, 2001). In a monolayer assay DIF-1 induces isolated amoebae to become stalk cells and it directly activates transcription of *ecmA*, a gene encoding an extracellular matrix protein (Williams et al., 1987). Cap-site distal elements of the *ecmA* promoter (the ecmO promoter region) are selectively utilised in, and provide a marker for, pstO cells and pstO/ALC (Early et al., 1993). Cap-site proximal elements of the *ecmA* promoter (the ecmA region) are selectively utilised in, and provide a marker for, pstA cells. Slugs formed by the dmtA− mutant, a strain defective in DIF-1 biosynthesis, show very little pstO differentiation (Thompson and Kay, 2000a) but this is now known to be mainly due to a buildup of an inhibitory synthetic intermediate; single mutants in the gene encoding the DIF-1 polyketide synthase (stlB), or dmtA−/stlB− double mutants, show a much less dramatic reduction in pstO differentiation (Saito et al., 2008). Thus DIF-1 is largely dispensable for pstO differentiation and is not required for pstA differentiation. There is some evidence that a polyketide other than DIF-1 may function as the pstA inducer (Serafimidis and Kay, 2005).

The only characterised molecular marker for pstB cell differentiation, ecmB-gal, utilises the entire *ecmB* promoter but it is inherently ambiguous; because *ecmB* is also expressed in the pstAB, upper cup and stalk cells (Ceccarelli et al., 1991). The DmtA− and PKS− mutants in DIF-1 biosynthesis display reduced *ecmB* expression in the lower cup and are also defective in formation of the outer basal disc (Saito et al., 2008). The cluster of neutral red staining pstB cells in the slug is greatly reduced in the PKS− strain but can be restored by addition of DIF-1 to the agar substratum. DIF-1 is therefore necessary, albeit to different degrees, both for normal levels of pstO and pstB differentiation. This raises an issue of specificity; how is a single molecule able to function in the differentiation of two different cell types? Resolution of this question will require a better understanding of the DIF-1 signalling mechanism(s) operative in the cell types.

Here we focus on DimB, a bZIP transcription factor that migrates to the nucleus and binds to two different sites in the *ecmA* promoter when cells are treated with DIF-1 (Zhukovskaya et al., 2006; Huang et al., 2006). In a dimB− strain the *ecmA* and *ecmB* genes are not inducible by DIF-1 in monolayer assay. In this study we analyse the cellular distribution of DimB in whole mounts of A × 2 slugs and culminants and correlate it with neutral red staining and *ecmB* expression patterns in different tissues. We show that pstB cells differ from all other cells in the slug, including pstO cells, in possessing a high relative concentration of DimB within their nuclei and we present evidence that DimB is a direct activator of *ecmB* transcription.

## Results

### In the slug DimB is highly nuclear enriched in the neutral red staining ventral cell population

**Fig. 1:**
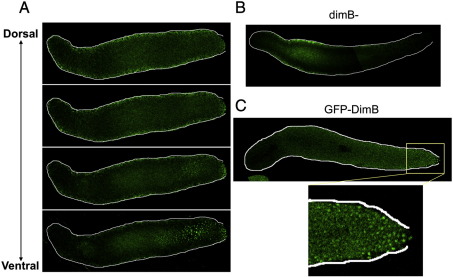
A) Whole mount DimB staining of a parental (A × 2) slug Serial confocal Z sections through a whole mount of a migratory slug, fixed, stained with anti-DimB antibody and then with Alexa 488-conjugated anti-rabbit antibody. B) Whole mount DimB staining of a dimB− slug As A) but a single confocal section of a dimB− slug from the ventral surface. C) An A × 2 slug expressing GFP-DimB A migrating dimB− slug expressing GFP-DimB visualised from its ventral surface by confocal microscopy.

DimB was analysed in whole mounts of slugs using a polyclonal antibody, raised against an internal peptide and affinity purified on the same sequence (Fig. 1A). When visualised by confocal microscopy there is weak, diffuse staining through most of the slug and more intense staining in the nuclei of a cluster of cells. As a control for the specificity of the antibody, slugs of a dimB− strain were analysed. They display a lower level of diffuse staining than the parent and there is no cluster of stained nuclei (Fig. 1B). The cluster is always located on the ventral surface of the slug but its position along the length of the slug is variable; it is generally located near the rear of newly formed slugs but after 4–5 h of migration it is often seen in more anterior positions.

**Fig. 2:**
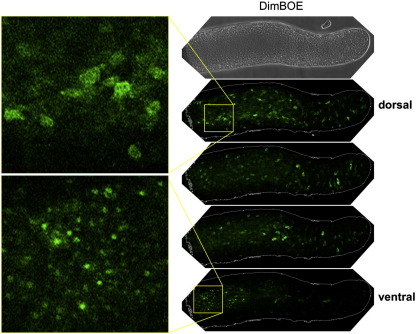
Whole mount of a dimB− slug expressing DimBOE and stained for DimB. DimB− slugs overexpressing dimB from its own promoter via the DimBOE construct were fixed, stained with anti-DimB antibody as in Fig. 1A. Confocal sections at different Z positions are shown. The top panel shows transmitted light.

We confirmed the existence of a cluster of cells with a high nuclear DimB concentration using two further analysis methods. Cells were transformed with a construct (GFP-DimB), that contains GFP as a reporter and which is expressed under control of the *dimB* promoter. Again, but now in living slugs, there is a posterior cluster of fluorescent nuclei (Fig. 1C). In the third approach cells were transformed with an untagged DimB over-expression construct (DimBOE), also under the control of the *dimB* promoter and in a dimB− background. The latter approach, using fixed samples stained with the DimB antibody, amplifies the immuno-staining signal considerably and such a confocal series is shown in Fig. 2. The DimBOE transformant was selected using G418 and therefore there is copy number variation between cells. This presumably accounts for the variable level of DimB protein in individual cells but, again, the only cells where DimB is nuclear-enriched form a cluster on the posterior ventral surface.

### During slug formation nuclear enrichment of DimB is initially widespread

**Fig. 3:**
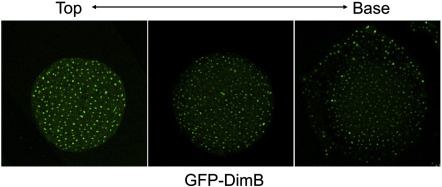
Serial confocal sections of an A × 2 mound stained for DimB. GFP-DimB cells were developed to the mound stage and visualised at different Z positions in the confocal microscope.

Using GFP-DimB we analysed the distribution of DimB during slug formation. At the tight mound stage, the first stage when a signal could be reliably detected, there are many cells with a nuclear enrichment in the most dorsal optical sections but there is apparently weaker nuclear enrichment in the more basal sections (Fig. 3). However, the sample was viewed from above using orthodox confocal microscopy so restricted light penetration probably accounts for the weaker fluorescence emanating from the more basal sections. As the tip extends, to form the first finger, nuclear enrichment in the apical regions is gradually lost but it is retained in the more basal regions (data not shown).

### The cluster of slug nuclei with a high concentration of DimB is pstB cells

**Fig. 4:**
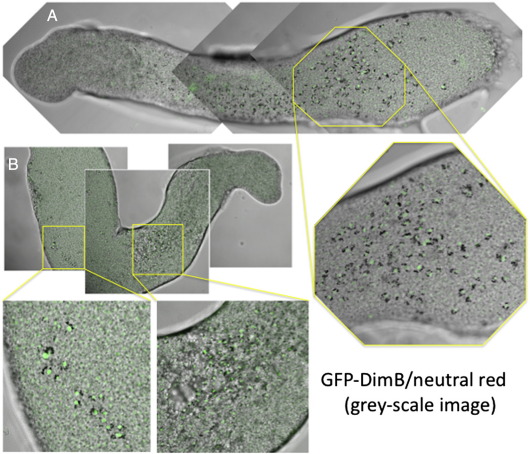
A × 2 slugs expressing GFP-DimB and stained with neutral red. GFP-DimB cells were stained with neutral red and allowed to migrate under unidirectional light until about 20 h of development. The ventral surfaces of two slugs (A and B) are visualised in the confocal microscope for both GFP fluorescence and transmitted light. The images were merged and the neutral red stained vesicles can be seen as dark granules, surrounding or flanking at one side the fluorescent nuclei.

Their ventral position, and variable location along the length of the prespore zone suggested that the slug cells with nuclear enriched DimB could correspond to the neutral red staining pstB cells identified by Dormann et al. (1996). This possibility was analysed using the GFP-DimB transformant. Cells were briefly incubated in neutral red, developed to the slug stage and analysed on a confocal microscope, using transmitted white light to visualise the neutral red stained cells (generating a grey-scale image) and fluorescence microscopy to visualise the GFP. The results of such an analysis are shown for two slugs where the DimB nuclear-enriched cluster is located at different positions along the AP axis (Fig. 4). The vital dye stained cells display large granular structures and there is an excellent correspondence with the presence in the same cell of a fluorescent nucleus. We conclude that the cells with high nuclear DimB are indeed the pstB cells.

**Fig. 5:**
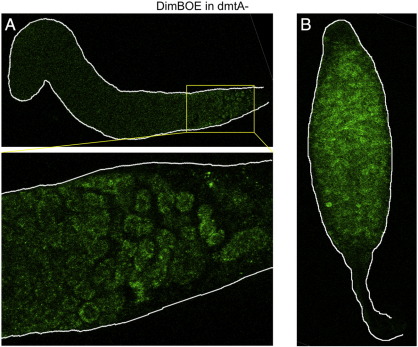
A) A slug of the dmtA− DIF biosynthesis mutant overexpressing DimB. DmtA− slugs overexpressing DimB were fixed and stained for DimB as in Fig. 1A. This is a ventral confocal section of a slug. B) A culminant of the dmtA− DIF biosynthesis mutant overexpressing DimB. As in A) except this is a culminant.

The above correspondence was further substantiated by analysis of a mutant with greatly reduced pstB cell differentiation. DmtA is the methyl transferase that acts at the last step in DIF-1 biosynthesis to create the active molecule. The null mutant for *dmtA* forms slugs with very little pstB cell differentiation, as monitored by neutral red staining (Saito et al., 2008). A dmtA− mutant strain was transformed with the DimBOE construct, developed to the slug stage and stained for DimB. There are, as expected, no cells with high nuclear enrichment of DimB (Fig. 5A, cf with Fig. 2).

### At culmination DimB is nuclear enriched in the lower cup and outer basal disc cells

**Fig. 6:**
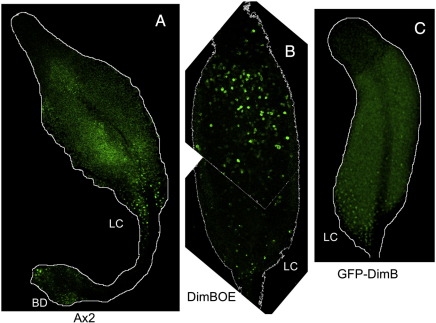
A) Whole mount DimB staining of an A × 2 culminant. B) Whole mount DimB staining of a DimB overexpressing culminant. C) An A × 2 culminant expressing GFP-DimB. Culminants of A × 2 (A) or DimB overexpressing cells (B) were fixed and stained for DimB as in Fig. 1A. (C) Culminants of GFP-dimB cells.

**Fig. 7:**
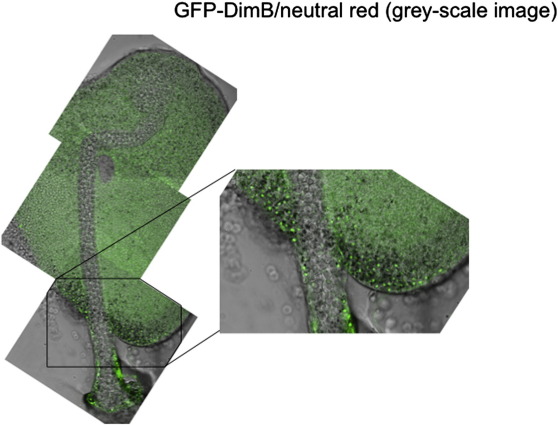
An A × 2 culminant expressing GFP-DimB and stained with neutral red. Culminants of GFP-DimB transformed cells stained with neutral red were visualised for GFP fluorescence and transmitted light as in Fig. 4.

At culmination the pstB cells move, some to become the lower cup and others the outer basal disc. The distribution of DimB was determined at the mid-culminant stage, using the same three methods employed for slugs: staining A × 2 parental structures with DimB antibody, staining the DimBOE strain with DimB antibody and direct visualisation of living structures expressing GFP-DimB. All three methods yielded the same result; there are cells with a high degree of nuclear enrichment in the lower cup and the basal disc (Figs. 6A, B and C; basal discs not shown for 6B and C). Vital dye staining of the GFP-DimB transformant supports this result; the cells in the lower cup and basal disc which stain with neutral red have high intra-nuclear levels of DimB (Fig. 7). Again in the dmtA DIF-1 biosynthesis mutant, transformed with the DimB over-expression construct and developed to the mid-culminant stage, there are no cells with high nuclear enrichment of DimB (Fig. 5B).

### A high level of DimB nuclear enrichment frequently correlates with *ecmB* expression

**Fig. 8:**
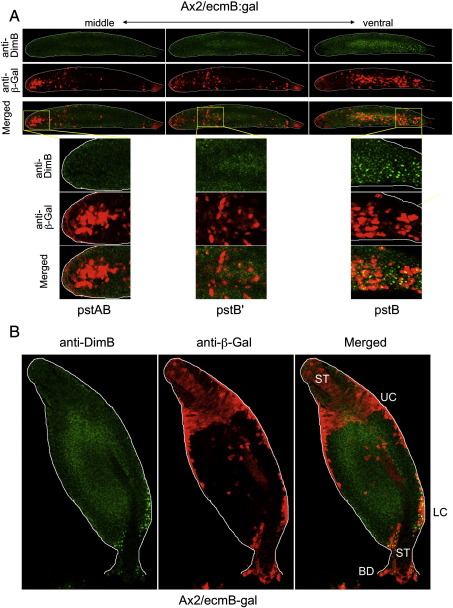
A. Serial optical sections though an A × 2 slug double-stained for ecmB-gal and DimB. Migrating A × 2 slugs transformed with ecmB:gal were fixed at around 20 h of development and double stained with anti-DimB and anti-β-galactosidase and then with Alexa 488-conjugated anti-rabbit antibody and Alexa 594-conjugated anti-mouse antibody. Confocal sections of a Z series extending from the middle to the ventral part of the slug and are expanded at three different positions along the length of the slug. B. An A × 2 culminant double-stained for ecmB-gal and DimB. As in A except that this is a single confocal section of a culminant.

The correlation between DimB nuclear enrichment and *ecmB* expression was analysed by immuno-staining of A × 2 cells transformed with ecmB-gal: detecting endogenous DimB using the DimB antibody and ecmB-gal using a β-galactosidase antibody. In the slug shown in Fig. 8 the cells with high intranuclear DimB are, as always, ventrally located. Their position on the AP axis is again, however, very variable between slugs. In this particular example they are located near the rear. Most of the cells in the region that express ecmB-gal have a high intranuclear concentration of DimB and those few that appear not to do so could be cells in an optical section that missed the nucleus.

Two other populations, both located dorsal to the pstB cells, express *ecmB* but do *not* display a high intranuclear concentration of DimB. There is the cone of pstAB cells located in the slug tip. Also, there is a loosely clustered group of cells, located at somewhat variable positions behind the pstAB cells but always close to the prestalk–prespore boundary (Fig. 8). We will, for the sake of clarity herein, term them pstB′ cells. Conversely, there are cells within the pstB region with a high intranuclear concentration of DimB that do not appear to express ecmB-gal (Fig. 8). We are unsure of the explanation for this. This particular example is a population selected in G418 so there could in principle be copy number variants in the population, but we obtain the same result when we analyse clones (data not shown). Perhaps there is heterogeneity within the population and the *ecmB* non-expressing cells accumulate DimB in the nucleus but require a separate trigger to induce expression.

**Fig. 9:**
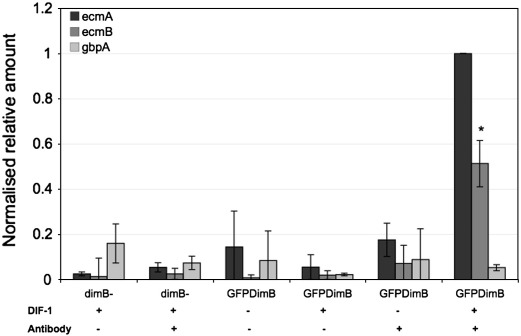
ChIP analysis of DimB binding to the *ecmB* promoter. Cells were incubated with or without DIF for 4 h and subjected to ChIP analysis as described in the Materials and methods section. The absolute recoveries from the procedure varied from experiment to experiment, (three independent experiments with triplicate Q-PCR analysis in each) but the induced signal for *ecmA* was always the highest. Therefore values are normalised to this and are shown with their Standard Deviations and with the Student's paired *T* test; applied to the *ecmB* analysis with and without DIF-1 and in samples immuno-precipitated from GFP-DimB transformant cells. As indicated by the asterisk the induction by DIF is significant with a P < 0.05.

The correlation between *ecmB* expression and nuclear enrichment of DimB persists into culmination; the lower cup and the outer basal disc show both features but DimB is not nuclear enriched in either the stalk or the upper cup (Fig. 9). At this stage there is an almost complete overlap between nuclear enrichment of DimB and *ecmB* expression, which argues strongly against there being any effect of copy number variation in these cells (see above).

### DIF-1 induces binding of DimB to the *ecmB* promoter

The correlation between DimB nuclear enrichment and *ecmB* expression would be most simply explained if DimB were to function as a direct transcriptional activator. This was tested by ChIP analysis using dimB− cells transformed with GFP-DimB. Cells were left uninduced or exposed to DIF-1 for 4 h and then subjected to ChIP using the GFP tag for immuno-purification. The negative controls for the precipitation were dimB− cells with no fusion protein and samples subjected to mock antibody precipitation. The negative control gene for the amplification was gbpA and the positive control for the induction was *ecmA* (Zhukovskaya et al., 2006). There is a DIF-dependent, antibody-dependent enrichment for both *ecmA* and *ecmB* with no significant enrichment for gbpA, as assayed using Q-PCR. In control, dimB− cells exposed to DIF-1 there is also no significant enrichment (Fig. 9). The highest signal was always with the *ecmA* primers and so the other results are normalised to this level. The two fold lower level of enrichment for *ecmB* may be the result of a weaker response of *ecmB* to DIF-1 by all cells in the population. Alternatively, it could reflect heterogeneity in the differentiated population; such that some cells in the population become *ecmA* expressing while a smaller fraction become *ecmB* expressing cells.

### In a dimB− strain neutral red staining of pstB cells and of the lower cup is very much reduced

**Fig. 10:**
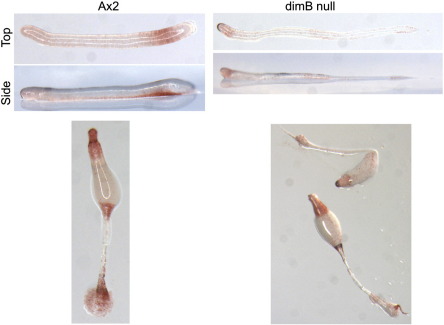
A × 2 parental and dimB− mutant slugs and culminants stained with neutral red. A × 2 and dimB− cells stained with neutral red were incubated under unidirectional light till 20 h of development then migrating slugs and culminants were visualised from the side by light microscopy.

In order to extend the above correlations by genetic analysis, vital dye staining was performed on a dimB null (dimB−) strain. Neutral red staining of parental A × 2 slugs revealed the expected pattern; staining in cells throughout the anterior, prestalk zone and a band of strongly stained cells, located at variable positions along the slug and closely apposed to the ventral surface (Fig. 10). In the dimB− slugs anterior staining is retained but the neutral red staining band is either absent or in some cases greatly reduced. In parental culminants there are neutral red stained cells in the apical region, lower cup and basal disc. In the dimB− strain the apical zone is stained, there is little or no basal disc tissue and lower cup staining is generally absent. There are, however, in some structures unstained, apparently undifferentiated, cells situated at the position normally occupied by the lower cup and at the base of the stalk tube.

### In slugs of a dimB− strain the cluster of cells with a high level of *ecmB* gene expression is absent and at culmination there is no *ecmB* expression in the vestigial lower cup

**Fig. 11:**
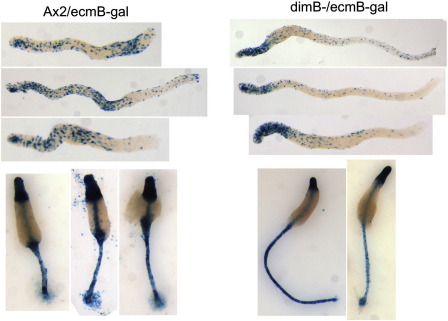
Enzymatic assay of ecmB-gal expression in A × 2 parental and dimB− mutant slugs and culminants. Migrating slugs or culminants of ecmB:gal transformants of A × 2 and dimB− were stained for β-galactosidase and visualised by light microscopy.

In our previous study of the dimB− strain *ecmB* expression was analysed by double enzymatic staining of transformants co-expressing ecmB-gal and psaA:gus (Zhukovskaya et al., 2006). The latter reporter was used to visualise the prespore cells. We concluded that pstB differentiation was normal in the dimB− strain. However, given the present results, we now believe that the strong prespore staining masked the true pstB cell population and that we mistakenly identified the pstB′ cells as pstB cells (Fig. 8). Hence we decided to re-analyse *ecmB* expression in the mutant using ecmB-gal alone. This revealed the expected staining pattern for pstB; a cluster of expressing cells located at variable positions along the prespore region of the slug (Fig. 9). In the dimB− strain there are pstB′ cells in the prespore–prestalk boundary region but there is no obvious cluster of expressing cells in the prespore region. In culminants there is staining in the upper cup and papilla but no staining in the position normally occupied by the lower cup (Fig. 11).

## Discussion

### A high intra-nuclear concentration of DimB is a correlate of pstB cell differentiation

PstB cells were originally identified by their elevated *ecmB* gene expression (Jermyn et al., 1996) and, in a parallel study, by their high level of staining with neutral red (Dormann et al., 1996). The proposed correspondence between the two populations was based solely on their apparent anatomical overlap. Here we find that there is an excellent correspondence between those cells with high intra-nuclear DimB and the neutral red stained cluster of cells on the ventral surface of the slug. Neutral red stains large acidic vesicles selectively located within prestalk cells and a model for anterior prestalk cell–prespore divergence is based upon a difference in vesicular pH (Gross, 2009). It is possibly therefore of mechanistic significance, for understanding the nuclear accumulation of DimB, that the pstB cells possess acidic vesicles. Many of the neutral red staining cells, with a high intranuclear concentration of DimB express *ecmB*. There are other *ecmB* expressing cells, the “pstB′ cells”, located close to the prestalk–prespore boundary but they display a relatively low intranuclear DimB concentration. Similar, anteriorly-located, *ecmB* expressing cells designated as pstB cells in a previous study (Keller and Thompson, 2008) are probably in reality also pstB′ cells.

A direct relationship between nuclear enrichment of DimB and *ecmB* expression is supported by genetic evidence. In a dimB− strain the neutral red staining mass of cells on the ventral surface is greatly reduced in size or in many cases absent. Concordantly, the cluster of *ecmB* expressing cells located at variable positions along the prespore region is also absent in the dimB− mutant slugs but the pstB′ cells remain. As expected, the two derivative structures from the pstB cells, the lower cup and outer basal disc, are much reduced or absent in the dimB− strain.

### How does DimB achieve and maintain a high relative concentration in pstB cell nuclei?

PstB cells, as identified by *ecmB* expression, differentiate at apparently random positions within the mound and then accumulate at the base as the tip is formed and extends (Jermyn et al., 1996). Their seemingly stochastic initial differentiation probably reflects heterogeneity in the cellular population that enters development. Nutritional status and cell cycle stage at the time of starvation are both believed to endow such heterogeneity, possibly by causing differences in sensitivity to DIF-1 (Thompson and Kay, 2000b and references therein). If there is inbuilt cellular heterogeneity it is not reflected in DimB nuclear accumulation at early developmental stages; we find no evidence for an initial high nuclear accumulation of DimB in a “proto-pstB” cell population. Instead, during slug formation, there is an accumulation of DimB in the nuclei of many of the cells in the upper part of the mound. It is only later, when the tip is considerably elongated that nuclear enrichment becomes largely restricted to the base. It would seem that only a sub-set of the cells that initially accumulate DimB in the nucleus go on to become pstB cells.

It is also mysterious how, in the migratory slug, DimB nuclear enrichment is maintained within the pstB cells despite their continuous, turbulent movement. The pstB cells are located adjacent to the ventral surface of the slug, so a DIF-1 gradient could in principle be responsible. The prespore cells are believed to be the source of DIF-1 and there is an anterior–posterior gradient of DIF-1 (Brookman et al., 1987), but there is no direct evidence for a dorso-ventral gradient. Even if there were such a DIF-1 gradient, this would beg the question of why all cells along the ventral surface do not become pstB cells. A related question is, how can one signalling molecule simultaneously stimulate, albeit with very different efficiencies, the differentiation of two distinct cell types: pstO and pstB cells? Answers to these questions will require fresh insights into the signalling pathways that utilise DimB.

### DimB and prestalk cell type divergence

Dissecting DimB function has been rendered more complex by the discovery that the effect of the dimB null mutation on developmental patterning is strain dependent. Analysis of a dimB− mutation in an A × 4 background showed reduced pstO differentiation in the slug, as monitored using ecmAO-gal (Huang et al., 2006). In contrast, there is a *higher* relative level of expression of ecmAO-gal in the pstO cells of A × 2-derived, dimB− slugs (Zhukovskaya et al., 2006). This suggests that in A × 2-derived slugs, but not in A × 4-derived, slugs, DimB functions as a repressor of pstO differentiation. Restricting discussion to A × 2, the strain in which the current analyses were made, then our results suggest a DimB concentration-dependent divergence of function. PstO cell nuclei contain a relatively low concentration of DimB and at such a concentration it functions as a repressor of ecmAO reporter gene expression. In pstB cell nuclei DimB is present at higher levels and at this concentration it acts as an inducer of *ecmB* gene expression.

If the above proposition is correct some signalling component other than DimB must serve as the activator of pstO differentiation in A × 2 cells. This could be DimA, the dimerisation partner of DimB, or it could be the Myb family transcription factor MybE (Fukuzawa et al., 2006). Both are required for *ecmA* expression in DIF-1 monolayer assay and for correct pstO cell differentiation in the slug. One feature of DimA, which argues against its involvement, is that it requires DimB for its accumulation in the nucleus (Huang et al., 2006); therefore a dimB null strain should, in effect, also be a dimA null. MybE is a better candidate but there is, as yet, no evidence of its regulation by DIF-1. A third transcription factor, that is DIF-1 regulated, the GATA factor GtaC is not required for *ecmA* inducibility in monolayer assay nor for correct pstO differentiation in the slug (Keller and Thompson, 2008). While it is not, therefore, a candidate for pstO activator it is required for normal levels of basal disc formation and so would appear to be involved in pstB cell differentiation.

### DimB and the control of *ecmB* transcription in monolayer assay

In monolayer cells DIF-1 induces transcription of *ecmA* and genetic analysis indicates that DimB is essential for the process (Huang et al., 2006b). The rapidity of induction suggests that DIF-1 acts as a direct inducer and delineation of binding sites in the promoter and ChIP analysis both further support the notion that DimB functions as a direct transcriptional inducer (Zhukovskaya et al., 2006). This striking difference from normal A × 2 development, where DimB acts a repressor rather than an activator of *ecmA*, could result from additional signalling inputs present only within a multicellular environment.

Another possible twist to this conundrum is provided by the suggestion that monolayer cells incubated in DIF-1 selectively may differentiate as pstB cells (Keller and Thompson, 2008). Without a marker specific for pstB cells this cannot be tested directly but one of the defining features of pstB cells is their high relative *ecmB* expression and low relative *ecmA* expression (Jermyn et al., 1996). Since pstB cells appear to be relatively inefficient at transcribing the *ecmA* gene during normal development (and if the suggestion by Keller et al. is correct) it seems possible that slug and monolayer pstB cells use a different signalling circuitry than is utilised for *ecmA* transcription in pstO cells. In this case a radical difference in DimB dependency for ecmO expression could be readily accommodated.

As would be predicted from the above hypothesis, in the case of *ecmB* the results for monolayer assay and normal development are concordant; DimB behaves genetically as an activator and ChIP analysis suggests that, under monolayer induction conditions at least, it is a direct transcriptional regulator (N. B. presumably because the fraction of pstB cells is very low attempts at ChIP analysis using slugs were inconclusive, unpublished data). This re-orients the focus of attention in studying DIF-1 signalling, from *ecmA* to *ecmB*. The promoter of the *ecmB* gene can be bisected into a cap-site distal region that directs expression only in upper cup cells and a proximal region, of 877nt, that directs strong expression in the stalk and much weaker expression in lower cup cells and outer basal disc cells. Positively and negatively acting regulatory elements that direct expression in the stalk have been mapped within the 877nt region and binding activities identified (Ceccarelli et al., 1991; Harwood et al., 1993; Ceccarelli et al., 2000). However, the regulatory regions that direct expression in the pstB cells, and that presumably bind DimB, are entirely unknown. Mapping them may yield a marker that is totally specific for pstB cells and that would be an invaluable tool for further investigations of *Dictyostelium* pattern formation.

## Materials and methods

### Cell culture, transformation, development, neutral red staining and enzymatic staining

A × 2 cells (Gerisch isolate) and derivative strains were grown axenically, transformed and subjected to development as described previously (Yamada et al., 2010). For neutral red staining vegetative cells were suspended in KK2 (20 mM K_2_HPO_4_/KH_2_PO_4_ pH 6.2) containing 0.0075% neutral red and then washed in KK2 until there was no residual colour in the wash. Cell type-specific expression of lacZ reporter constructs in developing structures was assayed either by enzymatic staining (Dingermann et al., 1989) or they were in some cases fixed and stained with an anti-β-galactosidase antibody (Yamada et al., 2010). GFP-DimB expression in developed structures was observed under silicon oil using a confocal microscope (Dormann and Weijer, 2006).

### Generation of a DimB antibody and immuno-staining

The rabbit anti-DimB polyclonal antibody was generated, using the C-terminal 10 amino acids of DimB, and purified as described (Zhukovskaya et al., 2006). For whole mount staining, structures developed on JA filters (Millipore, Billerica, MA, USA) sitting on agar plate were fixed with 50% methanol and then with 100% methanol. After rehydration, structures were stained with anti-DimB and with the indicated secondary antibody in PBS containing 5% BSA.

### Gene manipulation

For construction of DimBOE a dimB genomic region containing the gene and 2.1 kb of 5′ upstream sequence was PCR amplified using oligonucleotides: 5′-AGTCTAGACAAGATTGAAAACCAGACCCCC-3′ and 5′-GACTCGAGTTATTGTCTCGAAGGTTGTTGTTGG-3′, cloned into a *Dictyostelium* vector carrying a neomycin resistance cassette using XbaI and XhoI and transformed into dimB− cells. Transformants were selected with 50 μg/ml of G418 and cloned to obtain multicopy overexpressors of dimB. GFP-dimB was created by amplifying 2.1 kb upstream of the dimB gene, using oligonucleotides: 5′-AGTCTAGACAAGATTGAAAACCAGACCCCC-3′ and 5′-ACTAGTTTCTGTAATTTTTATTGATATTGTAAATTTTGAATTAAATG-3′ and fusing the product upstream of GFP, which was in turn fused to the DimB coding region to give a translational fusion protein. The fusion gene was cloned into a vector bearing a blasticidin resistant cassette and transformed into dimB− cells. Transformants were selected with 10 μg/ml blasticidin and cloned before use.

### ChIP analysis

GFP-DimB transformant cells, created in a dimB null background, and control dimB null cells were developed to the loose aggregate stage and mechanically disaggregated by syringing. They were induced by shaking for 4 h at 4 × 10^6^ cell/ml in KK2 containing 2 mM cAMP, either with or without 100 nM DIF-1. Chromatin samples were prepared and analysed essentially as described in Zhukovskaya (2006). Immunoprecipitation was carried out in the presence or absence of a polyclonal GFP antibody (Roche Diagnostic, Mannheim, Germany) at 4 °C overnight. Then 50 ul of Protein G coupled magnetic beads (Invitrogen Ltd, Renfrew, UK) was added to each sample and incubated for 4 h. DNA was recovered and samples were heated overnight at 65 °C to reverse cross-linking. After treating with Proteinase K (100ug/ml) and RNAse (10ug/ml) for 1 h at 37 °C, DNA was purified on QIA Quick-Spin columns (Qiagen, UK). QPCR was performed with inmunoprecipitated DNA or total genomic DNA using the following primers *ecmA*; forward TATTGCGTAATGGTTTTGCGGTC and reverse GGATTGTCGATCATATTTGATTAGTG (region − 453 to − 417); *ecmB forward* ATTTAGTAGCAAGTGGGTTAGTGTGGG and reverse TTACAAATCATACTATAATGATACGGGG (region − 826 to − 712) and (as a control) *gbpA forward* CATATAACACGATTGTAAAAAAAAAC and reverse GTTTGTTTAAAATTGAGTGTGGGTTG (region − 731 to − 583).
